# Short-Term Exercise Improves Not Only Muscle Strength But Also Lung Capacity, Endurance, and Quality of Life in Postmenopausal Vertebral Osteoporosis

**DOI:** 10.5152/ArchRheumatol.2025.11032

**Published:** 2025-06-23

**Authors:** Ülkü Uçar, Sibel Çubukçu Fırat, Tülay Ozdemir, Ersin Lüleci

**Affiliations:** 1 Department of Rheumatology, Antalya Research and Training Hospital, Antalya, Türkiye; 2 Department of Physical Medicine and Rehabilitation, Akdeniz University Medical Faculty, Antalya, Türkiye; 3 Department of Chest Diseases, Akdeniz University Medical Faculty, Antalya, Türkiye; 4 Department of Radiology, Akdeniz University Medical Faculty, Antalya, Türkiye

**Keywords:** Exercise, menopause, muscle strength, osteoporosis

## Abstract

**Background/Aims:**

The aim was to investigate the effectiveness of exercise on muscle strength, lung capacity, spinal mobility, endurance, and quality of life (QoL) in postmenopausal osteoporosis patients without vertebral fractures.

**Materials and Methods:**

This study was conducted with 41 postmenopausal osteoporosis patients (aged 45-65 years) without osteoporotic fractures. Patients were randomized into 2 groups. The patients in the exercise group (EG) were given an exercise regimen (breathing, stretching, relaxation, balance, and strengthening exercises) 3 times a week for 8 weeks at the department. Patients in the control group were kept on their current medical treatment. A Cybex Isokinetic Dynamometer and a Saunders digital inclinometer were used to assess back extensor muscle strength and spinal mobility. Pulmonary function tests were performed with a Jaeger spirometer. “Timed loaded standing” method, Quality of Life Questionnaire of the European Foundation for Osteoporosis 41 (QUALEFFO-41) and Short Form Health Survey 36 (SF-36) were used to evaluate the impact of exercise on back endurance and QoL respectively.

**Results:**

Baseline demographic and clinical characteristics were similar between the 2 groups. At the end of the study, statistically significant improvements were noted in the EG’s back extensor strength and endurance compared to baseline values (*P* < .05). Vital capacity, forced vital capacity, forced expiratory volume during the first second, maximal mid-expiratory flow rate, maximum inspiratory pressure measurements, and scores for QoL (physical function, mental function sub-scores, and total QUALEFFO-41 score, physical function, and vitality sub-scores of SF-36) were also significantly improved in the EG (*P* < .05). Spinal mobility of patients remained unchanged at the end of the study for both groups (*P* > .05).

**Conclusion:**

Muscle strength, trunk endurance, pulmonary functions, and QoL are known to be affected in postmenopausal osteoporosis patients. The findings supported that significant improvements can be achieved in these parameters even with appropriate short-term exercise in fracture-free periods.

## Introduction

**Main Points**

- Osteoporosis affects muscles, lung capacity, spinal mobility, and quality of life.
- In this study short-term exercise significantly improved back muscle strength, back endurance, respiratory functions, and Quality of life in postmenopausal osteoporosis patients without vertebral fractures.
- Longer exercise programs are required to increase spinal mobility.
- Preventive exercise is beneficial to reduce physical, functional, and psychosocial impairments related to osteoporosis before fractures occur in postmenopausal women.

Osteoporosis (OP) was described as “progressive systemic skeletal disease characterized by low bone mass and microarchitectural deterioration of bone tissue, with a consequent increase in bone fragility and susceptibility to fracture” by the World Health Organization in 1994.^1^ Osteoporosis is emerging as a serious public health issue as life expectancy increases. In Türkiye, the prevalence rates for men and women 50 or older are 22.2% and 27.2%, respectively.^2,3^

The significance of OP arises from fractures and related complications ranging from no pain to loss of function and even death.^4^ One in 5 men and 1 in 3 women with OP experience 1 or more osteoporotic fractures over their lifetime.^5^ After postmenopause, age and estrogen deficiency-related excessive trabecular bone resorption results in vertebral OP. Deformities in the osteoporotic spine and decreased back extensor muscle strength may develop a kyphotic posture. The presence of thoracic kyphosis has been associated with reduced rib mobility and alterations in respiratory function, even in patients without evidence of vertebral fractures.^6,7^ Consequently, OP may cause a decline in the individual’s physical and social functions and general well-being.^8^ Exercises that develop muscular strength and balance are essential for treating and preventing OP.^9^ In a systematic review, it was suggested that exercise is efficacious in improving the bone mineral density (BMD) and quality of life (QoL) of postmenopausal osteoporotic women without fractures.^5^ Nevertheless, a paucity of data remains regarding the impact of exercise on OP during the fracture-free interval, as the majority of research concentrates on OP in conjunction with fractures.

It was hypothesized that exercise may have a favorable effect in pre-fracture period patients with postmenopausal OP regarding musculoskeletal, pulmonary, and psychosocial aspects. In this study, the aim was to search the effects of an 8-week supervised exercise treatment on back extensor muscle strength, spinal mobility, trunk endurance, lung capacity, and QoL in postmenopausal osteoporotic women without fractures.

## Materials and Methods

### Participants

Fifty-four postmenopausal osteoporotic women between the ages of 45 and 65 without a history of osteoporotic fractures were recruited from the outpatient clinics of the institution’s physical medicine and rehabilitation department. Patients with spinal or hip BMD T-score of −2.5 or below were included using DXA (Dual-energy X-ray Absorptiometry) measurements on the lumbar spine (L1-L4) and femoral neck. Anteroposterior and lateral thoracic and lumbar spinal radiographs of the patients were evaluated for the presence of a fracture. A 20% or more decrease in the anterior, middle, or entire corpus height of the T4 and L5 vertebra was considered a fracture. Patients with malignancy, cardio-pulmonary disease, congenital chest and spinal deformity, neuropsychiatric disease, and present or past smokers were excluded from the study. Secondary causes of OP, musculoskeletal or cognitive barriers to exercise, inability to cooperate in pulmonary function tests, and a history of regular physical activity or involvement in any exercise program in the preceding 6 months were not eligible. The study’s design, the exercise regimen, the potential risks of osteoporosis, and the anticipated outcomes were all thoroughly described. Two patients with asthma, 1 patient with a history of thyroid malignancy, 3 patients with cardiovascular disease, and 4 patients with unwillingness to participate were excluded from the study. A total of 44 women were randomized to one of the 2 study groups: the exercise group (EG) or the control group (CG). The sequentially numbered, opaque, sealed envelope method was used for randomization. Of the 22 patients in the CG, 3 were excluded from the study because 1 developed Crohn’s disease and the other 2 could not be reached. Consequently, the final sample consisted of 41 postmenopausal women (22 patients in EG, and 19 patients in CG). Researchers and participants were not blind to allocation. Every participant underwent baseline and 8-week assessments. The study was approved by the Local Ethics Committee for Medical Research of Akdeniz University (Approval date: March 6, 2007, Decision no: E.01608), and written informed consent was obtained from all participants.

### Exercise Program

The exercise program was conducted for 8 weeks, including 3 sessions per week (totaling 24 sessions), each lasting 60 minutes, in the department’s exercise hall. All participants were encouraged to attend all the sessions. The same physiotherapist supervised and monitored the patients throughout the study. The program consisted of 10 minutes of warm-up and stretching exercises, followed by 10 minutes of bodyweight exercises, 30 minutes of balance, back extensor strengthening, abdominal, and upper and lower extremity muscle exercises, concluding with 10 minutes of breathing and cool-down relaxation exercises. For the back extensor strengthening, patients were instructed to lie in the prone position with a cushion under their abdomen for support. They were asked to keep 1 leg straight and lift it off the ground while extending the opposite arm diagonally forward. In addition, patients were instructed to perform opposing arm and leg raises in a hands-and-knees position. The exercises were performed for 3 sets of 8 repetitions during the first 4 weeks, and for 3 sets of 10 repetitions during the following 4 weeks For upper extremity strengthening, the shoulder press exercise was performed using 1 kg dumbbells. For lower extremity strengthening, a leg lift and hold exercise was performed in a hands-and-knees position, extending the leg backward while maintaining a 90° knee flexion. In the child’s pose, stretching exercises targeting the spinal musculature, hip flexors, thigh muscles, and ankle joints were implemented. While lying on their back, patients were instructed to hold their knees with both hands and pull them towards their chest to stretch the lower back and hip area. Additionally, hip flexor stretches were also included in the program. For balance training, patients were instructed to stand on 1 leg with their arms extended to the sides and maintain the position for as long as they could. This exercise was repeated for 3 sets on each leg. For breathing exercises, patients were instructed to perform diaphragmatic breathing for 3-5 minutes. While lying supine, they were advised to position 1 hand on the upper chest and the other on the abdomen, just below the rib cage. Patients were instructed to inhale slowly through the nose, ensuring that the abdomen rises against the hand, while keeping the hand on the chest as still as possible. Subsequently, patients were instructed to exhale slowly through pursed lips. The CG continued the current medical treatment without any exercise recommendations.

### Study Measures

The patients’ demographic characteristics were recorded. Age, body mass index, menopause duration, OP treatment and duration, chronic diseases, number of gestations, duration of breastfeeding, femur, and L1-L4 vertebra DXA scores were questioned**. **A “Digi-walker SW-401” pedometer was used to record the patient’s physical activity levels before the study. The daily number of steps was determined by taking the 3-day average of the value obtained at the end of the third day. In lateral dorsolumbar radiographs, the kyphosis angle was evaluated using the Cobb method.

### Back Extensor Muscle Strength

At the beginning and end of the 8-week exercise program, all patients’ trunk extensor muscle strength was measured and recorded with the CYBEX brand isokinetic exercise device in the department’s rehabilitation unit. Muscle strength measurement was performed isometrically while standing in the 20° flexion position. One trial was composed of 3 repetitions with a 5-second contraction and a 10-second rest, and peak rotation moment values were recorded. The peak torque measurements (in Newton meters) were used to assess muscle strength.

### Spinal Mobility

At the beginning and end of the study, participants’ spinal mobility was measured with a Saunders digital inclinometer according to the manufacturer’s recommendations. The 3 landmarks used in upright standing position are; the C7-T1 interspace, the T12-L1 interspace, and the S1-S2 sacral midpoint. Then, lumbar flexion and thoracic flexion angles were measured at maximum flexion, and lumbar extension and thoracic extension angles were measured and recorded at maximum extension.

### Pulmonary Capacity

The participants’ pulmonary function tests (PFTs) were performed with a Jaeger spirometer in the chest diseases department of the institution, in an upright sitting position, and with the nose clamp closed. A flow-volume curve was drawn, and vital capacity (VC), forced expiratory volume during the first second (FEV1), FEVl/forced vital capacity (FVC), maximal mid-expiratory flow rate (FEF 50), and maximum voluntary ventilation (MVV), peak expiratory flow (PEF) were recorded. The maximum inspiratory (MIP) expiratory pressure (MEP) was measured. All measurements were conducted by the same technician before and at the end of the intervention.

### Endurance

We used the timed loaded standing method which is a reliable and valid assessment tool of combined trunk and arm endurance in OP.^10^ The test calculates how long someone can stay upright while holding a two-pound dumbbell in each hand, with their elbows extended and their arms at a 90° shoulder flexion. The measurements were performed at the baseline and the end of the study.

### Quality of Life

The effect of exercise on QoL was assessed with the short form health survey 36 (SF-36) and quality of life questionnaire of the European Foundation for Osteoporosis 41 (QUALEFFO-41) self-administered questionnaire reliable and valid for Turkish patients.^11^ Eight dimensions of health—physical function, physical role difficulties, social function, pain, vitality, mental health, emotional role difficulties, and general health—were scored from 0 (worst) to 100 (best) using the SF-36. Questions in QUALEFFO-41 are divided into 5 categories: pain, mental performance, mental capacity, overall health perception, social functioning, and the ability to perform physical functions. These 5 domains can be assessed separately or as part of a total score. Every score is given on a scale of 0 to 100, with 100 being the worst possible score and 0 being the best.

### Statistical Analysis

The data were analyzed using IBM SPSS 21.0 software (IBM SPSS Corp.; Armonk, NY, USA). The normality of data was assessed using the Kolmogorov-Smirnov test. The results were presented as median (25%-75%), or frequency and percentage. Intragroup comparisons were made using the Wilcoxon signed-rank test. Additionally, the Mann-Whitney *U* test was used to compare the 2 groups. For measurements in the 8th week, percent changes were calculated according to the baseline measurement. These percent changes were compared using the Mann-Whitney *U* test for 2 groups. Correlation evaluation between parameters was made using Pearson and Spearman’s correlation analysis. A value of *P* < .05 was accepted for statistical significance. Post hoc power analysis was conducted based on the primary outcome measure, back extensor muscle strength. According to the post hoc power analysis performed using GPower 3.1.9.7 (Franz Faul, University of Kiel, Germany) with an effect size (d) of 0.90 and an α error probability of 0.05, the calculated power (1-β error probability) was 0.80.

## Results

Forty-one women completed the study (22 patients in EG, 19 in CG). Three drop-outs (7%) were seen in CG which was considered to be relatively acceptable. Table 1 describes the characteristics of the 41 participants. There was no significant difference between the 2 groups regarding the baseline variables. Exercise group patients were encouraged to attend all the sessions. The minimum attendance rate was 83.3%. Table 2 displays the subjects’ back endurance, back extensor muscle strength, QUALEFFO-41 and SF-36 results, and spinal mobility measurements at the beginning of the study. There were no statistically significant differences in these variables among groups at the baseline. Only physical role limitations and vitality scores of SF-36 were significantly higher in the EG. On the other hand, the EG group had significantly better results in terms of back endurance and back extensor muscle strength compared with the CG at the end of 8 weeks (*P* < .05) (Table 3). When PFTs, MVV, and MEP values were compared, no significant difference was found between the 2 groups pre and post-exercise pulmonary parameters (*P* > .05). Only the MIP value was significantly higher in CG than in the EG at the baseline. In the CG, there was no significant difference between the measurements made at the beginning and end of the study (*P* > .05). A significant increase was shown in the VC, FVC, FEV1, FEF 50, and MIP levels (*P* < .05) of EG at the end of 8 weeks. When the differences were compared between groups, VC, FVC, FEV1and MIP measurements were significantly increased in the EG compared to CG. Lung capacity measurements and the comparison of differences among groups at the end of the 8th are summarized in Table 4. No significant correlation could be detected between the kyphosis angle and lumbar and femur T scores, VC, FVC, FEV1, MIP, and MEP measurements in both groups at the baseline.

The comparisons of differences in endurance, back extensor strength, and QoL among EG and CG are summarized in Table 5. A significant increase in back extensor muscle strength and endurance was shown in EG compared to CG levels (*P* < .001 and *P* < .05 respectively).

**Figure 1.**
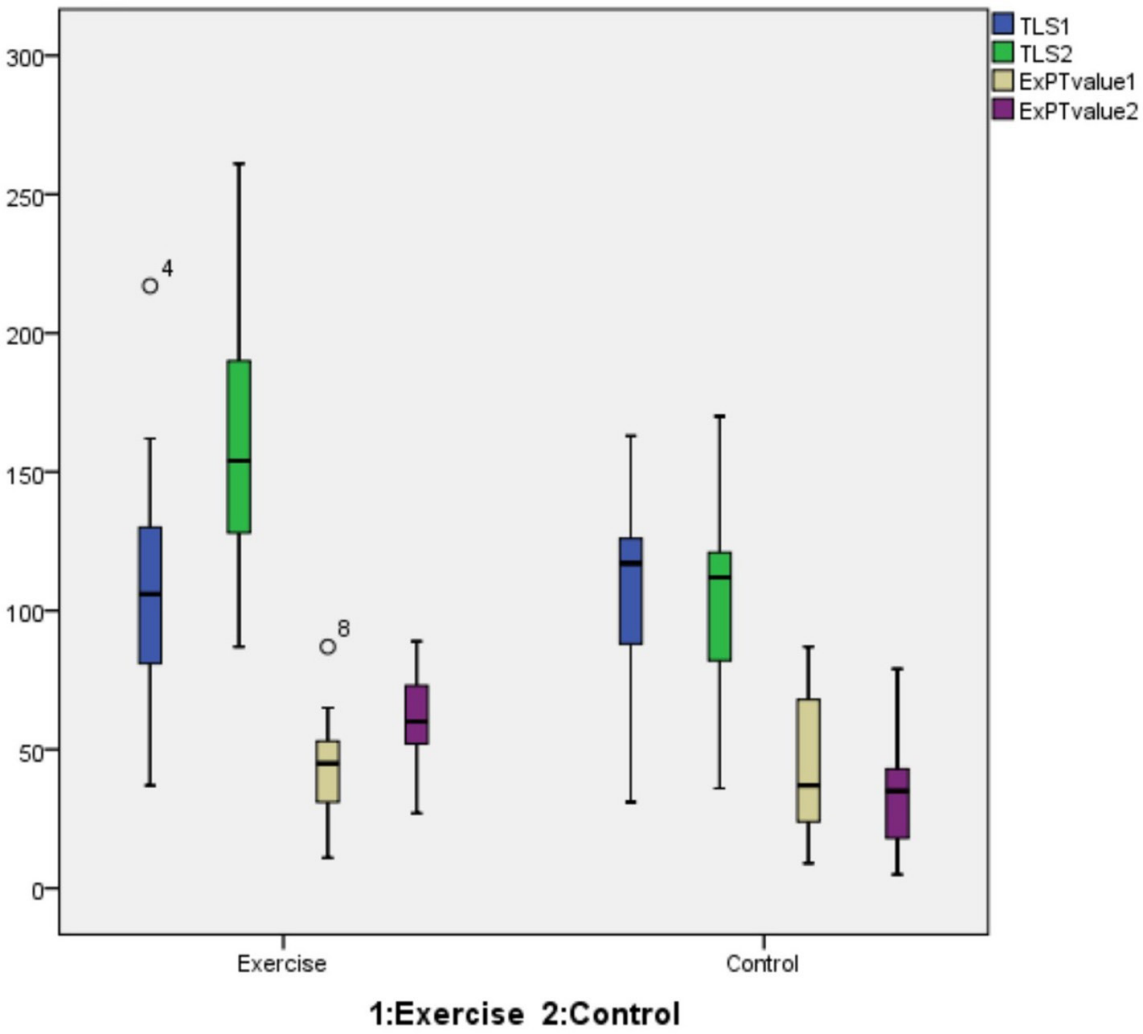
Comparison of trunk endurance and back extensor muscle strength of both groups between the baseline and 8 weeks. 1 stands for measurement at the baseline and 2 stands for measurement at 8 weeks. ExPT, back extensor strength; TLS, Timed loaded standing score (trunk endurance).

In the EG, significant improvements in back endurance and back extensor muscle strength were detected at 8 weeks, compared with baseline (*P* < .001) (Figure 1). Also, some domains of QUALEFFO-41 (total score, physical function, and mental health subgroups), and SF-36 (physical function and vitality domains) (*P* < .05) were significantly better at 8 weeks, compared with baseline (Table 6). Although there was an improvement in pain, social function, and general health scores of SF-36, it was not statistically significant (*P* > .05). When the differences were compared, physical function, social function domains of QUALEFFO-41, and role limitations due to physical health domain of Sf-36 were better in EG compared to CG (*P* < .05) (Table 5). When the spinal mobility measurements of the EG before and after the exercise program were compared, no significant difference was shown (*P* > .05). As no significant changes were observed in spinal mobility either within or between groups after the intervention, this parameter was not included in the comparative analysis. When the baseline and 8th week measurements of back extensor strength, endurance, QUALEFFO-41, SF-36, and PFT scores of CG were compared, no statistically significant difference could be found between measurements (*P* > .05).

## Discussion

The present study revealed that an 8-week supervised exercise program resulted in significant improvements in back extensor strength, trunk and arm endurance, as well as components of QoL and pulmonary functions in women with postmenopausal OP. Among the parameters evaluated, only spinal mobility has not improved after the 8-week intervention.

The first critical interpretation of the advantages of back exercise regimens for OP patients was conducted in 1982.^12^ Since then, several studies have demonstrated the importance of exercise in the treatment of OP.^13^ Studies have shown a connection between back extensor muscle strength and spinal BMD.^14^ An exercise program for at least 8 weeks, 3 or 4 times per week, regardless of the occurrence of vertebral fractures, seems to be essential.^15^ In this regard, an 8-week exercise regimen that comprised warm-up, stretching, balance, breathing, and strengthening exercises 3 times weekly for 8 weeks was designed.

There is limited data about the effectiveness of exercise on patients without a prior history of fractures, as most studies focus on rehabilitating OP after a fracture occurs. In postmenopausal OP patients, Sinaki et al^16^ found a strong negative correlation between thoracic kyphosis, the number of vertebral fractures, and back extensor muscle strength. A randomized controlled study, including patients with postmenopausal OP with fractures, showed that a 1-month group-adapted supervised exercise program was more effective in improving spinal pain, functional mobility, and QoL than a home-conducted program.^17^ A systematic review supports the beneficial effects of exercise on postmenopausal OP without fractures in terms of BMD, QoL, pain, balance, and functional status.^5^ This study demonstrated a significant improvement in extensor muscle strength in the EG at the end compared to the pre-exercise state, which is consistent with prior studies. Muscle weakness, posture disorder, and associated vertebral fractures may cause back pain and fatigue in OP. Limited data exist on the physiological basis of fatigue and the diminished endurance of the back muscles in OP. In this study, at the end of the 8-week exercise program, a significant increase in back and arm endurance was found in the EG in parallel with the improvement in isometric muscle strength. Also, the endurance of the EG was significantly better than that of the CG at 8 weeks. Moreover, the comparative analysis revealed that the EG showed a significant improvement in back extensor strength and endurance, highlighting the positive impact of the exercise intervention on trunk musculature.

Spinal mobility is also affected in OP. Tsauo et al^18^ reported that spinal mobility was significantly lower in OP compared to osteopenic patients.^18^ In this study, the spinal mobility measurements of the EG and CG were similar. It was thought that the homogeneity of the patients, in terms of factors such as muscle strength, BMD, physical activity levels, and age, that may affect spinal mobility, led to similar results. Hongo et al^19^ reported a significant improvement in QoL and back extensor muscle strength after a 4-month low-intensity home exercise program in the EG. However, there was no significant difference in spinal mobility.^19^ Similarly, in this study, in the EG, no improvement could be achieved in spinal mobility measurements after the intervention. It was concluded that the 8-week exercise period and the intensity of the exercises may not be sufficient to affect mobility at the level of the intervertebral joints.

Menopause-associated changes can also affect lung functions. A population-based survey study showed that postmenopausal women and women in the climacterium have a more rapid deterioration in lung function than non-menopausal women.^20^ In addition to hormonal influences, increased kyphosis affects pulmonary functions in OP.^21^ The extent of impairment in pulmonary function correlates with the size of the kyphosis angle; larger angles indicate worse lung capacity.^22^ It was reported that 19% of osteoporotic women without vertebral fractures had increased kyphosis, leading to a decrease in FVC.^23^ Not surprisingly, no correlation could be shown between the kyphosis angle and respiratory parameters, as the cases were selected from patients without vertebral fractures whose thoracic kyphosis angles were within normal limits.

Lombardi et al^23^ reported that osteoporotic patients with vertebral fractures had lower VC and FEV1 values than both healthy controls and those without fractures. They showed decreased VC in patients with a kyphosis angle of 55° and above. In the QoL assessment with SF-36, no significant difference was found between the groups.^23^ In a more recent systematic review, researchers found that kyphosis in OP is associated with certain pulmonary function impairments, particularly in VC.^24^ Regarding previous data, PFTs, MIP, and MEP measures are often unaffected in the initial stages of OP; on the other hand, patients who do not have vertebral fractures may have reduced respiratory muscle endurance. Çimen et al^6^ compared 88 patients with vertebral OP without spinal fracture with 54 healthy individuals and showed no significant difference between the 2 groups in PFTs, MIP, and MEP measurements. However, the MVV value in the OP group was significantly lower. They concluded that respiratory muscle endurance may decrease by different mechanisms in OP patients without fracture.^6^ Another study emphasized that a decrease in VC might occur in 19% of OP patients without spinal fractures.^7^ In this study, significant enhancements in some parameters of flow rate, volume, and pressure measurements were achieved in the EG. Maximum voluntary ventilation and MEP measurements showed a non-significant increase. Through between‐group comparative analysis, it was found that the EG showed significant improvements in VC, FVC, FEV1, and MIP than the CG, underscoring the efficacy of the exercise. It may be speculated that the decreased chest expansibility in OP may cause imbalanced respiratory muscle functioning even in fracture-free periods and can be improved with targeted training.

In addition to physical symptoms, patients with OP may experience challenges with self-care and everyday life tasks. Research has indicated that women with osteoporotic spinal fractures had a poorer QoL than those without fractures. According to a recent meta-analysis, there was a positive correlation between QoL and the BMD of the lumbar vertebra and femoral neck. On the other hand, QoL was inversely correlated with the degree of fragility fracture.^25^ In a review, compared to the healthy controls, OP patients without fractures had worse scores on the sub-scores of SF36 and QUALEFFO-41.^26^ Papaioannou et al^27^ reported that a 6-month home exercise intervention improved the QoL in OP patients with at least 1 spinal fracture. Few studies have shown that OP reduces QoL without a fracture.^28^ Similarly, this study showed that 8 weeks of supervised exercise resulted in significant improvement in QUALEFFO-41 and SF-36 scores. Furthermore, it was hypothesized that engaging in social activity during a group exercise session under supervision instead of home-based might have an extra beneficial effect on patients’ psychosocial well-being.

There are some limitations in this study. While these findings are promising, the small sample size represents an important limitation; Further longitudinal and prospective studies are needed in larger samples to confirm the results and improve generalizability. Secondly, due to the lack of a follow-up assessment, the maintenance of improvements over time and the benefits of fracture risk are unknown. Also, as a healthy CG was not included, the study does not give an idea as to whether these parameters are impacted in individuals with osteoporosis compared to healthy individuals. The drop-out of 3 patients (7%) in the CG may also be considered as a limitation. Against this, high compliance was observed in the EG. Another limitation of this study is the lack of balance assessment, as the study design did not include an evaluation of the effects of exercise on balance parameters. Incorporating balance assessments in future studies may provide a more comprehensive understanding of the impact of exercise on functional outcomes in this population Moreover, none of the patients withdrew due to complications from their exercise program, and no adverse effects were noted.

In conclusion, the 8‑week exercise program significantly improved back extensor strength and endurance, respiratory function (VC, FVC, FEV₁, MIP), and QoL in postmenopausal OP patients without vertebral fractures. Although spinal mobility did not change over this period, longer‐duration interventions may be needed to see benefits in this parameter. These findings underscore the value of early, preventive exercise regimens to alleviate the physical, functional, and psychosocial impairments of OP even in fracture-free periods.

## Figures and Tables

**Table 1.** Baseline Characteristics of Subjects

	EG (n = 22) 53.7% Median (25%-75%)	CG (n = 19) 46.3% Median (25%-75%)	*P*
Number of steps/day Age BMI(kg/m^2^) Number of births Lactation (months) Kyphosis angle	7995.3 (5577-10148) 56.5 (54.5-60.2) 29.7 (24.9-30.9) 2 (1-3) 20 (9.75-30) 27.5 (24-37)	8391 (904.6-16837) 58 (57-60) 25 (23.6-27.8) 2 (2-3) 21 (8-36) 35 (30.5-37.5)	.714 .294 .158 .361 .733 .211
Menopause age Menopause duration(years) L1-L4 T score Femur T score OP treatment duration (years)	45 (43-48.2) 10.5 (7-17.5) −2.6 (−2.5- −3) −1.7 (−2.6- −1.4) 2 (1-3)	46 (42-50) 13 (7-16) −2.6 (−2.9- −2.6) −2.1 (−2.7- −1.3) 5 (2-3)	.400 .803 .834 .927 .097

BMI, body mass index; CG, control group; EG, exercise group; L1-4, lumbar 1-4; OP, osteoporosis.

**Table 2.** Comparison of Outcome Measures of the Exercise Group and Control Group at the Baseline

	EG (n = 22) Median (25%-75%)	CG (n = 19) Median (25%-75%)	*P*
Trunk and arm endurance (seconds) Back extensor stregth (N/m) Lumbar flexion (degrees) Lumbar extension (degrees) Thoracic flexion (degrees) Thoracic extension (degrees) QUALEFFO-41 score	106 (80-132.5) 45 (27.5-55) 46 (34.5-61) 10 (7-14.5) 67 (63.5-76.5) 41 (36-53.5)	117 (88-130) 34 (23-66) 49 (30-56) 13 (7-16) 70 (65-75) 48 (42-58)	.588 .758 .401 .464 .524 .147
Pain Physical functioning Social functioning General health Mental health Total QUALEFFO score	22.5 (15-45) 16.1 (8.1-23.9) 28.4 (19.5-44.1) 45.8 (25-58.3) 36.1 (22.2-45.1) 26.9 (21.3-38.4)	30 (10-45) 13.3 (2.9-26.5) 37.9 (17.9-61.8) 41.7 (16.7-58.3) 41.6 (16.7-58.3) 32.3 (21.3-39)	.834 .339 .327 .588 .359 .574
**SF-36**			
Physical functioning Role limitations due to physical health Bodily pain Social functioning Mental health Role limitations due to emotional health Vitality General health	73.5 (48.8-88.5) 100 (25-100) 77 (57.3-90) 75 (50-100) 64 (51-72) 66 (24.8-100) 60 (50-81.3) 67.5 (48.8- 81.3)	63.5 (48.8-88.5) 30 (0-81.3) 67 (44-80) 75 (48.8-90.3) 54 (35-68) 66 (0-100) 45 (25-63.8) 60 (38.8-66.3)	.470 .043 .101 .334 .127 .731 .018 .133

CG, control group; EG, exercise group; N, Newton; QUALEFFO-41, Quality of Life Questionnaire of the European Foundation for Osteoporosis-41; SF-36, Short Form Health Survey 36.

**Table 3.** Comparison of Outcome Measures of the Exercise Group and Control Group at the 8th Week of the Study

	EG (n = 22) Median (25%-75%)	CG (n = 19) Median (25%-75%)	*P*
Trunk and arm endurance (seconds) Back extensor stregth (N/m) Lumbar flexion (degrees) Lumbar extension (degrees) Thoracic flexion (degrees) Thoracic extension (degrees) QULAEFFO-41 score	154 (124-195.5) 60 (45.8-73) 44 (36.5-62) 9 (5.5-16.5) 68 (62-77) 35 (25.5-52)	115 (83.7-130.5) 35 (17.5-43.8) 62 (53-71) 12 (3-14.7) 75 (70-79) 32.5 (32-53)	.02 .01 .351 .947 .421 .749
Pain Physical functioning Social functioning General health Mental health Total QUALEFFO score	25 (8.7-35) 8.7 (4.7-17.3) 25 (9.9-91.9) 41.7 (25-40.9) 34.6 (25-40.9) 21 (15.4-28.9)	30 (17.5-45) 11.7 (4.4-30.1) 32.1 (17.8-51.1) 41.7 (25-58.2) 38.8 (26.4-59.7) 27 (20-42)	.424 .357 .056 .892 .167 .074
SF-36			
Physical functioning Role limitations due to physical health Bodily pain Social functioning Mental health Role limitations due to emotional health Vitality General health	82.5 (65-98.8) 100 (75-100) 77. 8 (67.6-90) 87.5 (67.5-97) 64 (49-72) 83.5 (0-100) 67.50 (55-80) 67.5 (50-80)	60 (34.7-85) 55 (0-100) 67 (51.5-96.8) 75 (62-97) 56 (45-66) 33 (0-100) 55 (27.5-78.8) 60 (46.3-75)	.062 .062 .036 .404 .262 .178 .140 .366

CG, control group; EG, exercise group; N, Newton; QUALEFFO-41, Quality of Life Questionnaire of the European Foundation for Osteoporosis-41; SF-36, Short Form Health Survey 36.

**Table 4.** Comparison of Pulmonary Function Tests, Maximum Voluntary Ventilation, Maximum Inspiratory Pressure, and Maximum Expiratory Pressure Values of Subjects At the Baseline and the 8th Week of Study

		EG (n:22) Median (25%-75%)	CG (n:19) Median (25%-75%)	*P*
VC (%)	Baseline	93.2 (81.4;101.2)	91 (74;106)	.845
	8th week	99.1 (89.9;112.7)	93 (76.5;103.5)	.108
	Δ	0.07 (0;0.16)	−0.01 (−0.02;0.05)	.015
	*P*	.001	.909	
FVC (%)	Baseline	92.9 (77.9;101.2)	90.1 (74;103)	.886
	8th week	97.8 (88.8;110.5)	92.5 (74.7;100.8)	.089
	Δ	0.07 (0.01;0.14)	0 (−0.02;0.07)	.010
	*P*	<.001	.925	
FEV1 (%)	Baseline	98.9 (89.6;109)	101 (82;115)	.865
	8th week	100.5 (93.7;119.7)	101.3 (83;110.7)	.356
	Δ	0.03 (0.01;0.09)	−0.01 (−0.03;0.04)	.009
	*P*	.002	.468	
FEV1 /FVC (%)	Baseline	114.5 (108.2;120.5)	119 (111;123)	.456
	8th week	112.3 (107.8;117.8)	117 (113.5;122.3)	.084
	Δ	0 (−0.06;0.04)	0 (−0.04;0.02)	.781
	*P*	.881	.528	
PEF (%)	Baseline	87.6 (77.1;96.9)	79 (75;90)	.295
	8th week	89.3 (75.9;103.3)	91 (73.3;97.5)	.442
	Δ	0.05 (−0.05;0.15)	0.04 (−0.05;0.16)	.872
	*P*	.223	.378	
FEF 50 (%)	Baseline	92.5 (69.5;108.5)	86 (75;112)	.754
	8th week	90.5 (77.8;125.2)	93.5 (73;118.8)	.668
	Δ	0.04 (−0.02;0.21)	0.05 (−0.09;0.16)	.455
	*P*	.034	.513	
MMEF (%)	Baseline	99.3 (69.9;113.7)	86 (79;105)	.272
	8th week	96 (77.7;114.6)	89 (71;116.8)	.802
	Δ	0.04 (−0.08;0.1)	0.04 (−0.11;0.14)	.965
	*P*	.485	.587	
MVV (%)	Baseline	76.7 (49.7;95.9)	81.5 (59.8;89.8)	.563
	8th week	83.1 (59.5;97.6)	76.5 (64.2;85.5)	.312
	Δ	0.06 (−0.1;0.33)	−0.03 (−0.15;0.08)	.271
	*P*	.346	.501	
MIP (cm H_2_O)	Baseline	20.7 (14.3;34.8)	35 (24;40.6)	.041
	8th week	31.8 (23.5;40.5)	30.5 (21.2;38.5)	.767
	Δ	0.39 (0.05;1.04)	−0.21 (−0.45;0.22)	.009
	*P*	.023	.363	
MEP (cm H_2_O)	Baseline	56.5 (35.9;79)	68.5 (55.5;83)	.165
	8th week	69.4 (51.7;81.5)	65 (50;79.3)	.487
	Δ	0.2 (−0.02;0.51)	0.01 (−0.33;0.23)	.108
	*P*	.098	.485	

cm, centimeters; FEF 50, maximal mid-expiratory flow rate; FEV1, forced expiratory volume during the first second; FVC, forced vital capacity; MEP, maximum expiratory pressure; MIP, maximum inspiratory pressure; MVV, maximum voluntary ventilation; PEF, peak expiratory flow; VC, vital capacity.

Δ, Percent Change = (8th week – Baseline)/Baseline.

**Table 5.** Comparison of Differences Between Groups At the 8th Week of the Study

	EG (n = 22) Median (25%-75%)	CG (n = 19) Median (25%-75%)	*P*
Δ Trunk and arm endurance (seconds)	0.44 (0.27-0.93)	−0.08 (−0.22-0.08)	<.001
Δ Back Extensor stregth (N/m)	0.38 (0.05-1.30)	−0.15 (−0.46-0.35)	.006
Δ QUALEFFO-41 score			
Δ Pain	−0.20 (−0.58-1.33)	−0.12 (−0.33-0.20)	.843
Δ Physical functioning	−0.47 (−0.60- −0.19)	0.33 (−0.38-1.43)	.035
Δ Social functioning	−0.31 (−0.17-0.05)	0.19 (−0.17-0.59)	.022
Δ General health	−0.06 (−0.29-0.18)	0.00 (−0.04-0.50)	.187
Δ Mental health	−0.15 (−0.34-0.004)	0.00 (−0.19-0.41)	.097
Δ Total QUALEFFO score	−0.24 (−0.33- −0.12)	0.02 (−0.15-0.16)	.05
SF-36			
Δ Physical functioning	0.00 (−0.25-1.00)	0.00 (−0.63-1.67)	.662
Δ Role limitations due to physical health	0.15 (0.00-0.38)	−0.12 (−0.33-0.00)	<.001
Δ Bodily pain	0.02 (−0.13-0.20)	0.07 (−0.10-0.49)	.539
Δ Social functioning	0.07 (−0.06-0.55)	0.00 (−0.14-0.24)	.438
Δ Mental health	0.00 (−1.00-0.50)	0.00 (−0.08-0.40)	.741
Δ Role limitations due to emotional health	0.00 (−1.00-0.50)	−0.34 (−1.00-0.00)	.180
Δ Vitality	0.08 (−0.06-0.29)	0.16 (−0.06-0.86)	.385
Δ General health	0.00 (−0.07-0.18)	0.04 (−0.14-0.33)	.789

CG, control group; EG, exercise group; N, Newton; QUALEFFO-41, Quality of Life Questionnaire of the European Foundation for Osteoporosis-41; SF-36, Short Form Health Survey 36.

**Table 6.** Comparison of Outcome Measures in the Exercise Group Among Baseline and the 8th Week of the Study

	Baseline Median (25%-75%)	8 weeks Median (25%-75%)	*P*
Trunk and arm endurance (seconds) Back Extensor stregth (N/m) Lumbar Flexion (degrees) Lumbar Extension (degrees) Thoracic Flexion (degrees) Thoracic Extension (degrees) QUALEFFO−41 score	106 (80-132.5) 45 (27.5-55) 46 (34.5-61) 10 (7-14.5) 67 (63.5-76.5) 41 (36-53.5)	154 (124-195.5) 60 (45.8-73) 44 (36.5-62) 9 (5.5-16.5) 68 (62-77) 35 (25.5-52)	<.001 .01 .492 .705 .379 .107
Pain Physical functioning Social functioning General health Mental health Total Qualeffo score	22.5 (15-45) 16.1 (8.1-23.9) 28.4 (19.5-44.1) 45.8 (25-58.3) 36.1 (22.2-45.1) 26.9 (21.3-38.4)	25 (8.7-35) 8.7 (4.7-17.3) 25 (9.9-91.9) 41.7 (25-40.9) 34.6 (25-40.9) 21 (15.4-28.9)	.393 .007 .052 .147 .031 .001
SF-36			
Physical functioning Role limitations due to physical health Bodily pain Social functioning Mental health Role limitations due to emotional health Vitality General health	73.5 (48.8-88.5) 100 (25-100) 77 (57.3-90) 75 (50-100) 64 (51-72) 66 (24.8-100) 60 (50-81.3) 67.5 (48.8- 81.3)	82.5 (65-98.8) 100 (75-100) 77. 8 (67.6-90) 87.5 (67.5-97) 64 (49-72) 83.5 (0-100) 67,50 (55-80) 67.5 (50-80)	.004 .077 .522 .131 .708 .414 .048 .436

N, Newton; QUALEFFO-41, Quality of Life Questionnaire of the European Foundation for Osteoporosis-41; SF-36, Short Form Health Survey 36.

## Data Availability

The data that support the findings of this study are available on request from the corresponding author.
